# Supplementary material for: Phylogenetic and Codon Usage Bias Analysis Based on mt-DNA of Cyphochilus crataceus (Coleoptera: Melolonthinae) and Its Neighboring Species
Source: Genes (Basel). 2025 Jan 21;16(2):111. doi: 10.3390/genes16020111 (PMC11854983; doi:10.3390/genes16020111)
Supplement: Supplementary file 1 [file genes-16-00111-s001.zip › genes-3361625-supplementary.pdf]

TableS1 The list of species analyzed in this study and their GenBank accession numbers

Number	GenBank accession number	Organism	Subfamily	Length(bp)	AT%
1	KX087240.1	*Aphodius foetens*	Aphodiinae	15907	76.3
2	MT872706.1	*Aphodius pedellus*	Aphodiinae	16325	76.9
3	KX087329.1	*Oxyomus sylvestris*	Aphodiinae	12839	75.8
4	MT548771.1	*Campsiura mirabilis*	Cetoniinae	16123	72.8
5	OM718873.1	*Clinterocera nigra*	Cetoniinae	17421	71.8
6	NC_065313.1	*Coenochilus striatus*	Cetoniinae	15480	73
7	OK012569.1	*Dicronocephalus adamsi*	Cetoniinae	18550	74.8
8	OK484300.1	*Dicronorhina derbyana*	Cetoniinae	16609	74.2
9	OK484299.1	*Eudicella quadrimaculata*	Cetoniinae	16690	73.7
10	OK484302.1	*Eudicella smithii*	Cetoniinae	16712	74.8
11	OK484301.1	*Eudicella tetraspilota euthalia*	Cetoniinae	18302	74.9
12	NC_063846.1	*Gametis jucunda*	Cetoniinae	17980	73.1
13	NC_063847.1	*Glycyphana fulvistemma*	Cetoniinae	16701	75.7
14	OK484303.1	*Goliathus goliatus*	Cetoniinae	18699	75.5
15	OK484304.1	*Jumnos ruckeri*	Cetoniinae	19468	71.8
16	OK484305.1	*Mecynorhina polyphemus*	Cetoniinae	16422	75.2
17	OK484306.1	*Mecynorhina torquata ugandensis*	Cetoniinae	17192	75.6
18	NC_030778.1	*Osmoderma opicum*	Cetoniinae	15341	71.7
19	MN418316.1	*Protaetia brevitarsis*	Cetoniinae	17783	75.1
20	OK484307.1	*Protaetia speciosa jousselini*	Cetoniinae	16955	75
21	NC_063849.1	*Trichius succinctus*	Cetoniinae	18358	75.3
22	OK484308.1	*Chalcosoma caucasus caucasus*	Dynastinae	19444	76.2
23	JX412731.1	*Cyphonistes vallatus*	Dynastinae	11629	67.5
24	OK484309.1	*Dynastes hercules hercules*	Dynastinae	17813	67.1
25	OQ998898.1	*Dynastes satanas*	Dynastinae	16973	69.6
26	NC_059757.1	*Eophileurus chinensis*	Dynastinae	16624	69.1
27	NC_065036.1	*Eupatorus gracilicornis*	Dynastinae	18391	75.5
28	NC_066495.1	*Eupatorus hardwickei*	Dynastinae	18494	75.4
29	NC_066494.1	*Eupatorus sukkiti*	Dynastinae	18445	75.5
30	OK484310.1	*Megasoma elephas elephas*	Dynastinae	16785	70.1
31	OK484311.1	*Megasoma mars*	Dynastinae	16983	69.9
32	OK484312.1	*Oryctes nasicornis*	Dynastinae	20396	70.3
33	ON764801.1	*Oryctes rhinoceros*	Dynastinae	17275	71.5
34	NC_062856.1	*Trichogomphus mongol*	Dynastinae	17377	77
35	OK484314.1	*Xylotrupes beckeri*	Dynastinae	18434	76.3
36	OK484313.1	*Xylotrupes beckeri intermedius*	Dynastinae	18567	76.3
37	OK484315.1	*Xylotrupes socrates tonkinensis*	Dynastinae	18660	77.1
38	OK484316.1	*Xylotrupes sumatrensis*	Dynastinae	19687	76.1
39	NC_036270.1	*Hydrochus carinatus*	Hydrochidae	16825	79.7
40	NC_065312.1	*Apogonia cf. basalis*	Melolonthinae	15226	77.2
41	NC_065311.1	*Apogonia splendida*	Melolonthinae	16728	74.8

42	NC_046890.1	*Cheirotonus gestroi*	Melolonthinae	16899	70.7
43	NC_023246.1	*Cheirotonus jansoni*	Melolonthinae	17249	65.6
44	MT548775.1	*Holotrichia diomphalia*	Melolonthinae	16293	73.6
45	MZ726798.1	*Holotrichia niponensis*	Melolonthinae	16851	74.3
46	MF997046.1	*Holotrichia oblita*	Melolonthinae	15968	72.7
47	MW874410.1	*Holotrichia parallela*	Melolonthinae	16975	75.2
48	KX087316.1	*Melolontha hippocastani*	Melolonthinae	15485	71.3
49	NC_068084.1	*Miridiba trichophora*	Melolonthinae	16284	74.2
50	NC_054285.1	*Polyphylla gracilicornis*	Melolonthinae	16793	68.9
51	MT548774.1	*Polyphylla laticollis*	Melolonthinae	16408	69
52	KF544959.1	*Polyphylla laticollis mandshurica*	Melolonthinae	14473	69.8
53	NC_013252.1	*Rhopaea magnicornis*	Melolonthinae	17522	75.6
54	NC_082142.1	*Sophrops peronosporus*	Melolonthinae	19948	72
55	OP963801	*Cyphochilus crataceus*	Melolonthinae	17946	71.82%
56	NC_065314.1	*Sophrops subrugatus*	Melolonthinae	16409	71.6
57	NC_087770.1	*Anomala aulax*	Rutelinae	16246	75.9
58	NC_069575.1	*Anomala corpulenta*	Rutelinae	16673	75.9
59	NC_082143.1	*Anomala rufiventris*	Rutelinae	17240	75.1
60	NC_065310.1	*Anomala russiventris*	Rutelinae	15601	74.7
61	NC_082144.1	*Callistethus plagiicollis*	Rutelinae	16870	73.3
62	MT548770.1	*Mimela splendens*	Rutelinae	15584	75
63	OP974626.1	*Popillia japonica*	Rutelinae	18406	75.7
64	NC_056126.1	*Popillia mutans*	Rutelinae	16192	75.9
65	KU739470.1	*Bubas bison*	Scarabaeinae	13466	75.1
66	KU739469.1	*Bubas bubalus*	Scarabaeinae	16035	77.6
67	KU739484.1	*Caccobius nigritulus*	Scarabaeinae	15039	80
68	MT548776.1	*Catharsius molossus*	Scarabaeinae	15036	77.3
69	KU739450.1	*Cheironitis hoplosternus*	Scarabaeinae	14924	75.8
70	NC_045923.1	*Copris tripartitus*	Scarabaeinae	15457	78.7
71	NC_039689.1	*Dichotomius schiffleri*	Scarabaeinae	14802	77.4
72	KU739497.1	*Digitonthophagus gazella*	Scarabaeinae	15302	76.4
73	KU739491.1	*Drepanocerus kirbyi*	Scarabaeinae	15780	79
74	KU739453.1	*Euoniticellus fulvus*	Scarabaeinae	15494	78.9
75	KU739490.1	*Euoniticellus intermedius*	Scarabaeinae	15578	77.6
76	KU739494.1	*Eurysternus caribaeus*	Scarabaeinae	15227	77.3
77	KU739455.1	*Eurysternus foedus*	Scarabaeinae	15366	76.6

78	KU739493.1	*Eurysternus hamaticollis*	Scarabaeinae	15428	78.7
79	KU739492.1	*Eurysternus inflexus*	Scarabaeinae	15766	78.4
80	KU739489.1	*Helictopleurus quadripunctatus*	Scarabaeinae	15265	78.7
81	MW759025.1	*Helictopleurus sicardi*	Scarabaeinae	14789	67
82	KU739468.1	*Heteronitis castelnaui*	Scarabaeinae	13441	76
83	KU739488.1	*Liatongus militaris*	Scarabaeinae	15832	79.4
84	KU739481.1	*Milichus apicalis*	Scarabaeinae	15823	78.8
85	KU739487.1	*Oniticellus egregius*	Scarabaeinae	15547	78.9
86	KU739467.1	*Onitis alexis*	Scarabaeinae	17501	78.1
87	KU739466.1	*Onitis falcatus*	Scarabaeinae	15763	78.4
88	KU739482.1	*Onthophagus alcyon*	Scarabaeinae	10725	78.4
89	KU739464.1	*Onthophagus baolocensis*	Scarabaeinae	16690	79.3
90	KU739472.1	*Onthophagus bicallosus*	Scarabaeinae	15361	76.5
91	KU739459.1	*Onthophagus bonasus*	Scarabaeinae	15730	78
92	KU739449.1	*Onthophagus cervicapra*	Scarabaeinae	14114	76.7
93	KU739458.1	*Onthophagus cf. jeannelianus*	Scarabaeinae	15654	79.6
94	KU739462.1	*Onthophagus cf. taurinus*	Scarabaeinae	15925	77.8
95	KU739447.1	*Onthophagus crassicollis*	Scarabaeinae	15370	79.4
96	KU739451.1	*Onthophagus cuniculus*	Scarabaeinae	15147	79.2
97	KU739483.1	*Onthophagus diabolicus*	Scarabaeinae	12098	75.3
98	KU739448.1	*Onthophagus falculatus*	Scarabaeinae	13980	78
99	KU739452.1	*Onthophagus fimetarius*	Scarabaeinae	16125	80.5
100	KU739461.1	*Onthophagus gracilipes*	Scarabaeinae	15342	79.2
101	KU739478.1	*Onthophagus haematopus*	Scarabaeinae	15301	78.5
102	KU739500.1	*Onthophagus longimanus*	Scarabaeinae	15374	77.4
103	KU739499.1	*Onthophagus nitidior*	Scarabaeinae	14329	78.8
104	KU739480.1	*Onthophagus nr. babirussa*	Scarabaeinae	15184	78.6
105	KU739477.1	*Onthophagus obscurior*	Scarabaeinae	13222	76.6
106	KU739456.1	*Onthophagus ochreatus*	Scarabaeinae	11488	82.2
107	KU739496.1	*Onthophagus pullus*	Scarabaeinae	15287	80.2
108	KU739498.1	*Onthophagus rhinolophus*	Scarabaeinae	15237	78.2
109	KU739476.1	*Onthophagus rorarius*	Scarabaeinae	15234	78.6
110	KU739471.1	*Onthophagus schwaneri*	Scarabaeinae	15581	78.9
111	KU739474.1	*Onthophagus vulpes*	Scarabaeinae	15884	79.2
112	KU739463.1	*Onthophagus yukae*	Scarabaeinae	16362	79.8
113	KU739473.1	*Phalops ardea*	Scarabaeinae	16248	77.7
114	KU739457.1	*Phalops barbicornis*	Scarabaeinae	13659	78.9
115	KU739495.1	*Phalops smaragdinus*	Scarabaeinae	15104	73.9
116	KU739460.1	*Scaptodera rhadamistus*	Scarabaeinae	15119	78.6
117	KU739486.1	*Tiniocellus sarawacus*	Scarabaeinae	15592	78.5
118	KU739485.1	*Tiniocellus spinipes*	Scarabaeinae	15177	79.2
119	KU739454.1	*Tragiscus dimidiatus*	Scarabaeinae	14206	78.7
120	JX412736.1	*Pleophylla* sp. PLE01	Sericinae	12579	72
121	MT872683.1	*Serica brunnea*	Sericinae	15152	74.1
122	MF997050.1	*Serica sp.* NS-2020	Sericinae	13815	73.3
123	MW829594.1	*Tetraserica leishanica*	Sericinae	10031	75.3
